# Supplementary material for: Accelerated and quantitative three-dimensional molecular MRI using a generative adversarial network
Source: Magn Reson Med. Author manuscript; Available in PMC 2024 May 1. (PMC9992146; doi:10.1002/mrm.29574)
Supplement: supinfo — Figure S1. Comparing the quantitative semisolid MT parameter maps from a GBM patient using N = 9 raw input images (A–D, M–P), N = 20 input images (E-H, Q-T), and CEST-MRF reference (I–L, U–X). Figure S2. A Monte Carlo simulation study comparing the quantification ability of GAN-ST trained on 9 or 30 inputs. Figure S3. Comparing the quantitative parameter maps obtained using GAN-ST with N = 9 raw input images (bottom row) and the respective output from CEST-MRF with N = 9 raw input images (center row), for the phantom data presented in Figure 2. Clearly, reducing the number of input images from N = 30 (top row) to N = 9 severely degraded the quantification performance for CEST-MRF, whereas GAN-ST resulted in better agreement with the ground-truth and the full length CEST-MRF. Table S1. Comparing Monte-Carlo based simulation of GAN-ST compound concentration quantification with 9 or 30 inputs. Table S2. Comparing Monte-Carlo based simulation of GAN proton exchange rate quantification with 9 or 30 inputs. [file NIHMS1860100-supplement-supinfo.pdf]

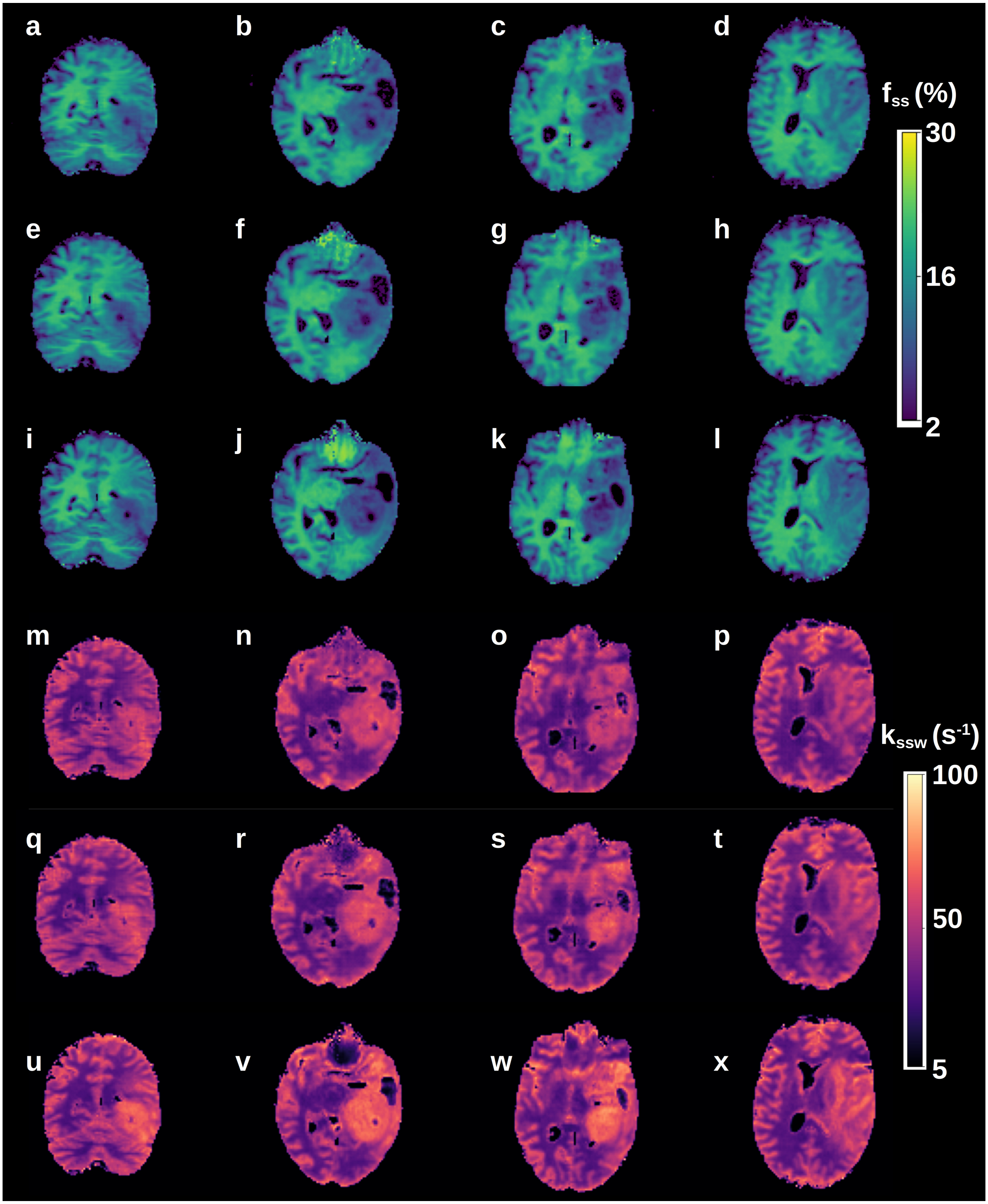

Supporting Information Figure S1. Comparing the quantitative semisolid MT parameter maps from a GBM patient using N=9 raw input images (a-d, m-p), N=20 input images (e-h, q-t), and CEST-MRF reference (i-l, u-x).

Monte Carlo Simulation Study (Supporting Information Figure S2, Tables S1 and S2)

A Monte Carlo simulation, examining the robustness of the acceleration and its dependence on target parameter range was performed. A simulated signal dictionary was generated where both the proton exchange rate and concentration of a CEST compound (L-arginine) was varied, yielding various parameter combinations (concentration: 10 - 120 mM, proton exchange rate: 100 – 1400 s^{-1}). Next, artificial images containing 6 circular ROIs were created with random combinations of the CEST parameters. These training image datasets were augmented by means of rotation and translation, similarly to the procedure performed in the phantom and in-vivo studies, with the same number of total images used. The corresponding raw MRF images were then calculated using the BM equations and used to train full-length (30-image input) and shortened (9-image input) GANs. A validation set was numerically created, composed of new parameter combinations that were not part of the training. The inference was repeated 10 times, each after injecting random white Gaussian noise of 53 dB to the test set images (as expected for in vivo CEST SNR, taken from Cohen et al., *Magn Reson Med*. 2023; 89: 233- 249). A comparison between the predicted compound concentration and proton exchange rate obtained by GAN-CEST with 30 or 9 images as input and the numerical ground truth is available in Supporting Information Figure S2, and Supporting Information Tables S1 and S2.

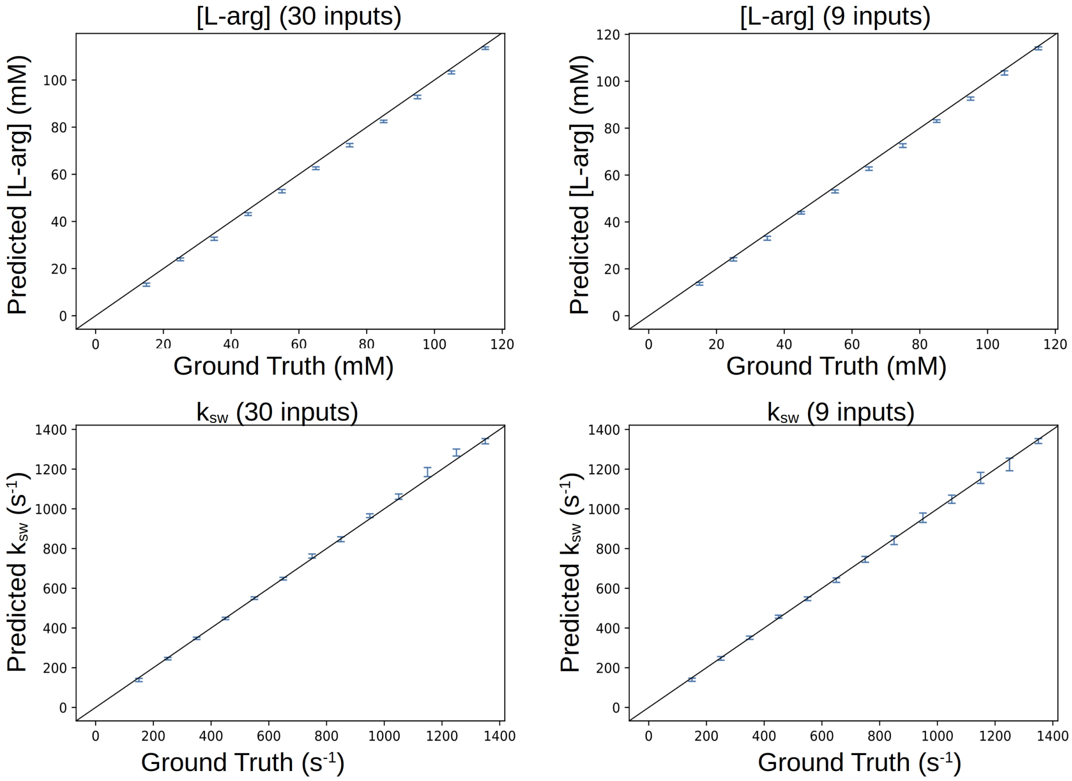

Supporting Information Figure S2. A Monte Carlo simulation study comparing the quantificaton ability of GAN-ST trained on 9 or 30 inputs.

Supporting Information Table S1. Comparing Monte-Carlo based simulation of GAN-ST compound concentration quantification with 9 or 30 inputs.

Ground Truth [L-arg]	Mean [L-arg]		STD [L-arg]	
	9 inputs	30 inputs	9 inputs	30 inputs
15	13.47	13.12	0.56	0.68
25	23.92	23.81	0.81	0.63
35	33.53	32.76	0.76	0.66
45	43.81	43.1	0.66	0.62
55	52.92	52.79	0.7	0.6
65	62.97	62.5	0.64	0.62
75	72.61	72.35	0.74	0.59
85	83.02	82.72	0.61	0.54
95	92.47	92.88	0.74	0.69
105	103.33	103.11	0.61	0.6
115	113.8	113.52	0.59	0.52

Supporting Information Table S2. Comparing Monte-Carlo based simulation of GAN proton exchange rate quantification with 9 or 30 inputs.

Ground Truth [L-arg]	Mean k_{sw} (s^{-1})		STD k_{sw} (s^{-1})	
	9 inputs	30 inputs	9 inputs	30 inputs
150	136.16	136.31	8.55	7.67
250	248.87	244.12	8.58	5.57
350	352.82	348.3	8.78	5.82
450	455.71	448.82	9.57	6.51
550	549.42	551.1	8.00	7.13
650	643.92	647.52	14.92	8.86
750	745.58	757.37	11.67	10.45
850	859.26	846.03	16.78	8.35
950	964.17	961.16	14.76	10.36
1050	1050.32	1059.63	24.74	14.12
1150	1146.16	1191.42	29.68	21.12
1250	1238.29	1285.34	29.6	16.47
1350	1340.61	1338.59	16.87	14.31

In nearly all cases, the standard deviation increased due to using 9 inputs instead of 30 for training the GANs (Supporting Information Table S1). However, it remained within a very reasonable range (concentration standard deviation < 0.81 mM and proton exchange rate standard deviation < 30 Hz). The largest standard deviation in estimating the concentration and proton exchange rates were obtained for the 9 input GAN case (at 25 mM and 1150 s^{-1}, respectively, which could be reasoned by the low sensitivity to small compound concentrations and the relatively low training examples available at the edge of the training range (1400 s^{-1}). Overall, an excellent correlation (Pearson's r > 0.95, p<0.0001) and ICC > 0.95 were obtained in all 4 cases analyzed in Supporting Information Figure S2.

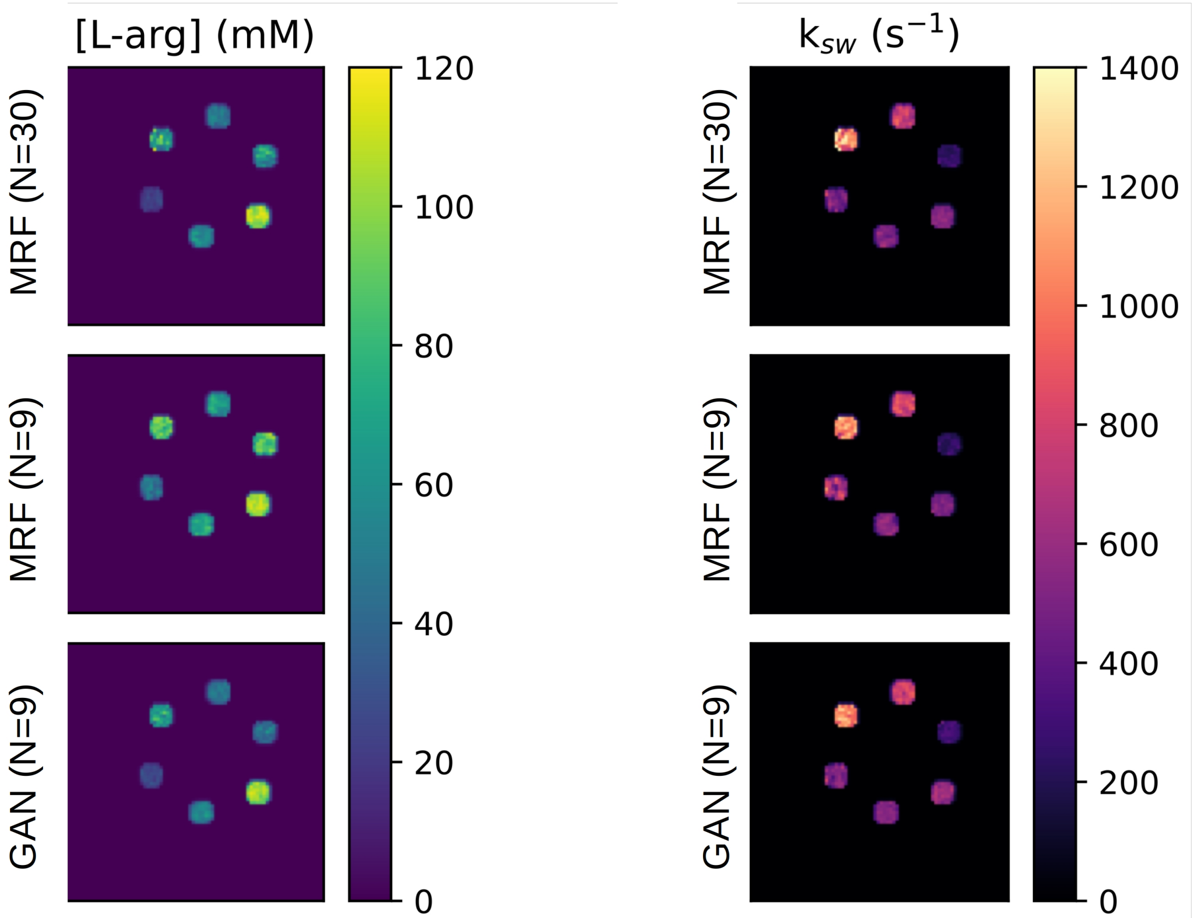

Supporting Information Figure S3. Comparing the quantitative parameter maps obtained using GAN-ST with N=9 raw input images (bottom row) and the respective output from CEST-MRF with N=9 raw input images (center row), for the phantom data presented in Figure 2. Clearly, reducing the number of input images from N=30 (top row) to N=9 severely degraded the quantification performance for CEST-MRF, whereas GAN-ST resulted in better agreement with the ground-truth and the full-length CEST-MRF.
